# Comparison of moderate-intensity continuous training and high-intensity interval training effects on the *Ido1*-KYN-*Ahr* axis in the heart tissue of rats with occlusion of the left anterior descending artery

**DOI:** 10.1038/s41598-023-30847-x

**Published:** 2023-03-06

**Authors:** Pouria Nori, Rouhollah Haghshenas, Younes Aftabi, Hakimeh Akbari

**Affiliations:** 1 Department of Sport Sciences, Faculty of Humanities, Semnan University, Semnan, Iran; 2 Associate Professor of Exercise Physiology, Department of Sport Sciences, Faculty of Humanities, Semnan University, Semnan, Iran; 3 Tuberculosis and Lung Diseases Research Center, Tabriz University of Medical Sciences, Tabriz, Iran; 4 Assistant Professor of Exercise Physiology, Department of Sport Sciences, Faculty of Humanities, Semnan University, Semnan, Iran

**Keywords:** Physiology, Diseases, Health care, Molecular medicine

## Abstract

Myocardial infarction (MI) affects many molecular pathways in heart cells, including the *Ido1*-KYN-*Ahr* axis. This pathway has recently been introduced as a valuable therapeutic target in infarction. We examined the effects of moderate-intensity continuous training (MICT) and high-intensity interval training (HIIT) on the axis in the heart tissue of male Wistar rats with occluded left anterior descending (OLAD). Thirty rats (age 10–12 weeks, mean weight 275 ± 25 g) were divided into five groups with 6 animals: Control (Ct) group, MICT group, rats with OLAD as MI group, rats with OLAD treated with MICT (MIMCT group) and rats with OLAD treated with HIIT (MIHIIT group). Rats performed the training protocols for 8 weeks, 5 days a week. HIIT included 7 sets of 4 min running with an intensity of 85–90% VO_2_max and 3 min of recovery activation between sets. MICT included continuous running at the same distance as HIIT with an intensity of 50–60% VO_2_max for 50 min. The expressions of *Ahr*, *Cyp1a1*, and *Ido1* were assayed by real-time PCR. Malondialdehyde (MDA) and Kynurenine levels, and AHR, CYP1A1, and IDO1 proteins were detected using ELISA. Data were analyzed using the ANOVA and MANOVA tests. Compared to the CT group, MI caused an increase in all studied factors, but only statistically significant (P < 0.05) for MDA and IDO1. With a greater effect of HIIT, both protocols significantly lowered the proteins expressions in the MIHIIT and MIMCT groups, compared with the MI group (P < 0.001). In healthy rats, only AHR protein significantly decreased in the MICT group compared to the Ct group (P < 0.05). HIIT and MICT protocols significantly reduced the gene and protein expression of *Cyp1a1* (P < 0.05) and *Ido1* (P < 0.01), and HIIT had a greater effect. In conclusion, both protocols were effective at reducing the levels of *Ido1*-Kyn-*Ahr* axis components and oxidative stress in the infarcted heart tissue and HIIT had a higher significant effect.

## Introduction

Myocardial infarction (MI) as a type of ischemic cardiovascular disease promotes adverse remodeling of the left ventricle by affecting cardiomyocytes and vascular cells, which altogether develop the first cause of morbidity and mortality worldwide^1^. These cellular changes involve a wide range of molecular pathways including the Kynurenine (KYN) metabolism and *Ido1*-KYN-*Ahr* axis^2^. KYN is a metabolite produced from the amino acid tryptophan (TRP) by the activity of tryptophan 2,3-dioxygenase and indoleamine 2,3-dioxygenase 1 (IDO1) and 2 enzymes, which regulates various immune and physiological responses via binding to and activation of the aryl hydrocarbon receptor (AHR)^3,4^. AHR is a ligand-activated transcription factor with myriad functions in health and diseases, which works upon binding to many intrinsic and extrinsic chemicals in physiological and immune responses and *Cyp1a1* upregulation is one of the main hallmarks of its activation^5,6^. *Ahr* knockout in a mouse model has been shown that it has highly crucial roles in maintaining the function, health, and physiological homeostasis of cardiac cells and tissues^7^. In MI, the *Ido1*-KYN-*Ahr* axis has paracrine effects on cardiomyocyte apoptosis and contractility and cardiac remodeling and function^2^. Furthermore, the levels of catabolites of this pathway in body fluids have been suggested as markers positively associated with MI and associated mortality^2^. KYN generation through IDO is markedly induced after MI and KYN metabolites may increase inflammation, oxidative stress, and apoptosis of smooth muscle cells and endothelial cells including cardiomyocytes^8–10^. It is shown that IDO1 activity has an inverse association with ischemic heart disease and therefore it has been introduced as a potential therapeutic target for this disease^11^.

Recently, it has been shown that exercise affects the *Ido1*-KYN-*Ahr* axis in different cells and tissues. Physical exercise has been shown to impact the KYN pathway (KP) in response to both acute and chronic exercise training stimuli and currently, it is accepted that exercise-induced KP may contribute to the prevention and treatment of chronic diseases^12,13^. Surprisingly, due to the effects of KP metabolites on skeletal muscle, adipose tissue, the immune system, and brain physiology, some researchers proposed that some of these metabolites could be suggested as exercise-induced myokines^14^. Also, other members of the *Ido1*-KYN-*Ahr* axis have been shown to interact with exercise metabolism. Pal et al., found that both acute and chronic endurance training may regulate NK cell function via the AHR/IDO axis^15^.

Increasing evidence confirmed exercise has improving effects on the cardiac function of MI patients^16,17^. All types of exercise can effectively inhibit skeletal muscle atrophy via reducing oxidative stress and protein degradation, increasing the antioxidant capacity, and regulating the growth factors expression^18^. However, there are different opinions about the impacts of exercise types on MI improvement. Cai et al. believe that in the early stages of MI, moderate-intensity exercise is the best choice to improve the outcomes for MI patients^18^. And Dun et al., reported that compared to moderate-intensity continuous training (MICT), supervised high-intensity interval training (HIIT) results in greater improvements in MI patients with metabolic syndrome^16^. Also, Moholdt et al. found that in MI patients aerobic interval training increases peak oxygen uptake more than usual exercise training^19^. Furthermore, different types of exercise affect KP in different ways. Joisten et al., reported that HIIT consistently led to greater effects than MICT on KP in persons with multiple sclerosis^20^. Currently, experimental studies aiming for a deeper understanding of cellular and molecular mechanisms underlying exercise interaction with the *Ido1*-KYN-*Ahr* axis in heart tissues of MI patients are still lacking. To fill some gaps in the current knowledge, here we conducted an in vivo investigation to compare the effects of MICT and HIIT on the axis in the heart tissue of rats with occlusion of the left anterior descending artery.

## Material and methods

### Experimental animals

Thirty male Wistar rats (age 10–12 weeks, mean weight 275 ± 25 g) were purchased from the Pasteur institute of Iran. Rats were kept in polycarbonate cages (Three rats per cage) on a 12 h light/dark cycle and a humidity of 65 ± 5% and a temperature of 25 °C and were provided food (rat chow) and water ad libitum. All experimental protocols were approved by the Committee on the Care of Laboratory Animal Resources, Semnan university of medical science (IR.SEMUMS.REC.1399.158) and were carried out following the Declaration of Helsinki, the ARRIVE guidelines and the Guide for the Care and Use of Laboratory Animals published by the US National Institutes of Health. Eighteen rats underwent surgery for occlusion of the left anterior descending (LAD). After evaluating and confirming MI using echocardiography, based on ejection fraction and fractional shortening the rats were divided into five groups including six members in each: Control group (Ct), a group treated with MICT, rats with occluded LAD (OLAD) as a model of MI, rats with OLAD and treated with MICT (MIMCT), and a group with OLAD and treated with HIIT (MIHIIT). Care of the animals was performed according to the European Convention for the Protection of Vertebrate Animals (ECPVA). The number of members in the groups was calculated following Charan and Biswas’s descriptions^21^.

### Occlusions of left anterior descending

After anesthesia of the animal using a combination of ketamine and xylazine, the surgical site on the animal’s chest was disinfected with 70% alcohol. After keeping the animal fixed on the operating desk, using an otoscope number 3 and a green angiocatheter, the animal was intubated and connected to a ventilator (inter med Bear) (inhaul to exhalation ratio of 1 to 2 and 80–90 breaths per minute with a volume of 8 ml). In the space between the third and fourth ribs, the chest was cut to a length of 10 mm. With this incision, the LAD vessel was identified as a bright red pulsating spike that flows in the middle of the heart wall from under the left atrium to the apex of the heart. The LAD vessel was closed with the help of 0.6 mm polypropylene suture 1–2 mm below the level of the tip of the left atrium and was completely closed by tying two knots at this point. Left ventricular anterior wall infarction was confirmed by sudden myocardial coloration (discoloration). An increase in ST was also observed after ligation. Then, the chest, muscle layers, and skin were sewn in three layers using 0.5 proline suture and the animal's skin was sutured with 0.3 proline suture. When the rats regained consciousness, they were removed from the ventilator. After 48 h, the rats were anesthetized again and with echo vivid7 probe 10 s (MHz), an echo was performed to determine MI. In addition, cefazolin and tramadol as antibiotics and analgesics were injected twice a day, 1 day before surgery and 3 days after surgery. Rats in the MI group underwent all surgeries without occlusion of the left coronary artery. Also, Ct group rats did not receive any intervention and were kept only in the laboratory.

### Exercise training

After evaluating and confirming MI and a week of rest MIHIIT, MICT, and MIMCT groups trained for three sessions per week for 2 weeks (each session 10–15 min at speed of 10 to 15 m per minute with a treadmill) to get acquainted with the training protocols. After 2 weeks of initial familiarization with the treadmill, the intensity of the training program was obtained in terms of VO_2_max and the relationship between VO_2_max and the speed and incline of the treadmill^22^. The aerobic capacity of the rats was evaluated after the initial warm-up. The test started with an initial speed of 6 m/min. The speed of the treadmill was increased every 2 min by 1.8–2 m/min until the rats reached the exhaustive stage. After obtaining the test speed and time, their average was calculated, and based on it, the protocol of training was designed. Then, the rats performed the two designed protocols of training based on previous reports^22–24^ for 8 weeks, 5 days a week with a slope of zero degrees (Table 1): (A) HIIT, which consisted of seven sets of four minutes running with the intensity of 85–90% VO_2_max and three minutes of recovery activation between sets with the intensity of 50–60% VO_2_max. (B) MICT, which consisted of continuous running in the same distance as HIIT with the intensity of 50–60% VO_2_max for 50 min. In both groups, warming and cooling down periods were performed for 5 min before and after the training with an intensity of 40% VO_2_max. The intensity.

**Table 1:** Protocol of exercise training.

	Warm-up	HIIT		MICT	Cooldown	Total time
		7 sets of 4 min	3 min			
Intensity VO_2_max	40%	85–90%	40%	60–65%	40%	60 min
1-2th week	5*	20–22	11	15–17	5	
3-4th week	5*	22–24	13	17–19	5	
5-6th week	5*	24–26	15	19–21	5	
7-8th week	5*	26–28	17	21–23	5	

^*^Meter/Minute: The intensity is expressed in meter/minute and the duration in minutes (min).

*HIIT* high intensity of interval training, *MICT* moderate intensity of continues training.

### Tissue sample preparation

Tissue resection was performed at the end of the eighth week and 72 h after the last training session. This was after performing anesthesia with CO_2_ gas and blood sampling from the heart. Then, heart tissue was extracted and after washing in physiological serum placed in microtubes and transferred to − 70 °C.

### Determination of malondialdehyde and KYN

For quantitative assay of KYN and malondialdehyde (MDA) in the heart tissue lysate, Kynurenine ELISA Kit, ZellBio GmbH (Cat. No: ZB-11203C-R9648; Germany) and MDA Assay kit (CAT No. ZB-MDA-96A; Germany) were used following the manufacturer’s instruction.

### Gene expression analysis

Total RNA was isolated from heart samples using TRIzol Reagent (Invitrogen, USA), treated with Dnase I, and quantified by NanoDrop (Thermo Fisher Scientific). RNA quality was determined by examining the 260/280 ratio > 1.8. A total of 1 µg RNA was then reverse transcribed to cDNA using the RevertAid First Strand cDNA Synthesis kit (Thermo Scientific) according to the manufacturer’s instructions. Expression of *Ahr*, *Cyp1a1*, and *Ido1*, was measured using specific primers produced by SinaColon, Iran (Table 2). Amplification was performed in ABI Prism 7500 sequence detection system; Life Technologies real-time RT-PCR device using the condition: initial denaturation (95 °C for 15 min for all genes); start of the cycle with denaturation (95 °C for 30 s for Gapdh, and 95 °C for 5 min for *Ahr*, *Cyp1a1*, and *Ido1*); annealing (55 °C for 30 s for Gapdh, 48 °C for 105 s for Ido1, 56 °C for 90 s for Cyp1a1and 60 °C for 90 s for *Ahr*); and extension (60 °C for 30 min for all genes). At the end of amplification cycles, reactions were given a final extension step at 60–95 °C. All data were analyzed with the ΔΔCt method and the expression of glyceraldehyde-3-phosphate dehydrogenase (*Gapdh*) was used as the internal standard.

**Table 2:** Primers.

Gene accession no	Sequences	Amplicon size (bp)	Annealing Tm (°C)	Ref
*Ahr*	FW: 5′-TCACTGCGCAGAATCCCACATCC-3′	186	60	^48^
NM_013149	RV: 5’-TCGCGTCCTTCTTCATCCGTTAGC-3’			
*Cyp1a1*	FW: 5′-GTCCCGGATGTGGCCCTTCTCAAA-3′	109 bp	56	
NM_012540	RV: 5′-TAACTCTTCCCTGGATGCCTTCAA-3′			
*Ido1*	Fw: 5′-GACTTCGTGGATCCAGAC-3′	277 bp	48	^49^
NM_02397.1	RV: 5′-TCTAAGGAGGAGAGGAAG-3′			
*Gapdh*	FW: 5′-GCCAAGGTCATCCATGACAAC-3′	600 bp	55	
NM_017008.4	RV: 5′-GTCCACCACCCTGTTGCTGTA-3′			

### Protein expression analysis by ELISA methods

ELISA kits ZellBio GmbH (Germany) were used for determining AHR (Cat. No: ZB-16349C-R), CYP1A1 (Cat. No: ZB-11059C-R9648), and IDO1 (Cat. No: ZB-10730C-R9648) levels in the heart tissue lysate following the manufacturer’s protocol.

### Statistical analysis

For data analysis, we used multivariate analysis of variance (MANOVA), which was previously reported as an efficient test for assessing multiple dependent variables simultaneously^25^. Data are presented as means ± standard deviation in the text and tables. The Pearson and partial correlation coefficients were used to measure the relationship between MDA, KYN, and protein levels. A one-way MANOVA was first performed to determine the effect of the factor ‘group’ on the latent variable. Since the result of the multivariate analysis was significant, univariate analyses were done to discover the effect of a significant factor on each indicator variable (AHR, CYP1A1, IDO1, KYN, and MDA) of the latent variable, which was followed by the Tukey’s post hoc test. Assumptions of analysis of variance were verified with the Shapiro–Wilk test, Levene’s test, Doornik-Hansen test, Box's M-test, and evaluation of the homogeneity of covariance matrices. The results were considered significant with P ≤ 0.05. All statistical computations were done using Stata version 16 (College Station, TX: Stata Corp LLC; 2019) and GraphPad Prism version 9.0.0 for Windows, GraphPad Software, San Diego, California USA, www.graphpad.com.

### Ethics approval

All experimental protocols were approved by the Regional Research Ethics Committee of Semnan University of Medical Sciences and Health Services (IR.SEMUMS.REC.1399.291) and were carried out following the Declaration of Helsinki, the ARRIVE guidelines and the Guide for the Care and Use of Laboratory Animals published by the US National Institutes of Health.

### Informed consent

Informed consent was obtained from animal’s owner involved in the study.

## Results

### Effect of MI and exercise on components of Ido1-KYN-Ahr axis

**Figure 1:**
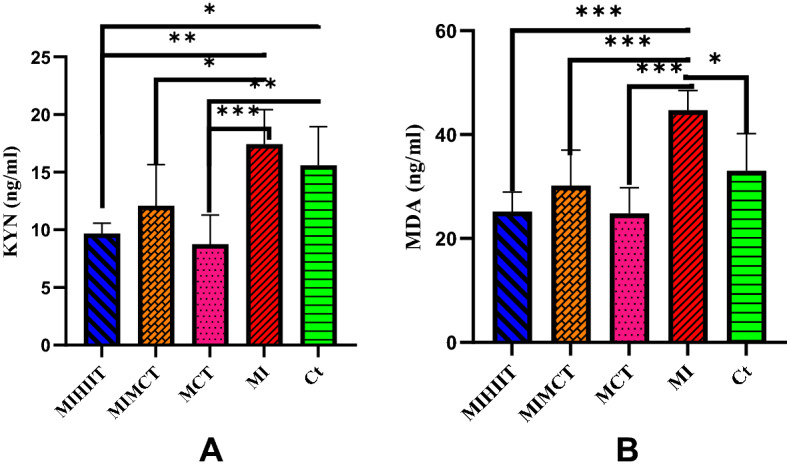
KYN (**A**), and MDA (**B**) levels in heart tissue of rats. MDA and KYN levels of the heart tissue were affected by the MI condition. Training had different effects on the treated groups. Abbreviations: *Ct* control group, *MI* the group with occlusion of the left coronary artery, *MICT* the group treated with moderate intensity of continuous training, *MIMCT* rats with left coronary artery occlusion treated with moderate intensity of continuous training, *MIHIIT* rats with left coronary artery occlusion treated with high-intensity interval training.

The results in Fig. 1 depict that MI increased the level of KYN, but this increase was not statistically significant compared to the Ct group. Both training protocols significantly reduced the level of KYN in the heart tissues of rats with MI (P < 0.05) and the effect of HIIT was greater than MICT (Table 3B). Also, MICT significantly reduced KYN in healthy rats (P = 0.003), which in general shows that exercise reduces KYN in heart tissue (Fig. 1A). MI significantly increased the level of MDA in the heart tissue of rats with MI (P < 0.011) and both HIIT (P < 0.001) and MICT (P = 0.001) significantly reduced its level.

**Table 3:** Relationships between variables and parameter estimates.

(A) The relationships between variables in liver tissue by Pearson and Partial correlation										
Variables	Correlation	AHR	IDO1	P-value	CYP1A1	P-value	KYN	P-value	MDA	P-value
			Coef		Coef		Coef		Coef	
AHR	r	1	0.62*	< 0.001	0.70	< 0.001	0.53	0.003	0.65	< 0.001
	r_p_		0.14	0.498	0.35	0.075	− 0.20	0.311	0.36	0.066
IDO1	r		1		0.81	< 0.001	0.79	< 0.001	0.67	< 0.001
	r_p_				0.47	0.013	0.49	0.010	− 0.08	0.678
CYP1A1	r				1		0.71	< 0.001	0.69	< .001
	r_p_						0.09	0.668	0.11	0.587
KYN	r						1		0.78	< 0.001
	r_p_								0.55	0.003
MDA	r								1	
	r_p_									

(B) Parameter estimates of variables in the five groups										
Group	Control	AHR		IDO1	CYP1A1		KYN		MDA	
MIHIIT	β	− 0.07		− 10.33	− 1.77		− 5.92		− 7.83	
	P-value	0.043		0.007	< 0.001		0.001		0.021	
MIMCT	β	− 0.07		− 2.75	− 0.20		− 3.50		− 2.83	
	P-value	0.027		0.440	0.439		0.043		0.381	
MICT	β	− 0.15		− 10.50	− 1.77		− 6.83		− 8.17	
	P-value	< 0.001		0.006	< 0.001		< 0.001		0.017	
MI	β	0.04		11.67	0.67		1.83		11.67	
	P-value	0.235		0.003	0.015		0.274		0.001	

*r* Pearson correlation,  *rp* Partial correlation, *Coef* Coefficient, *β* Parameter estimates.

**Figure 2:**
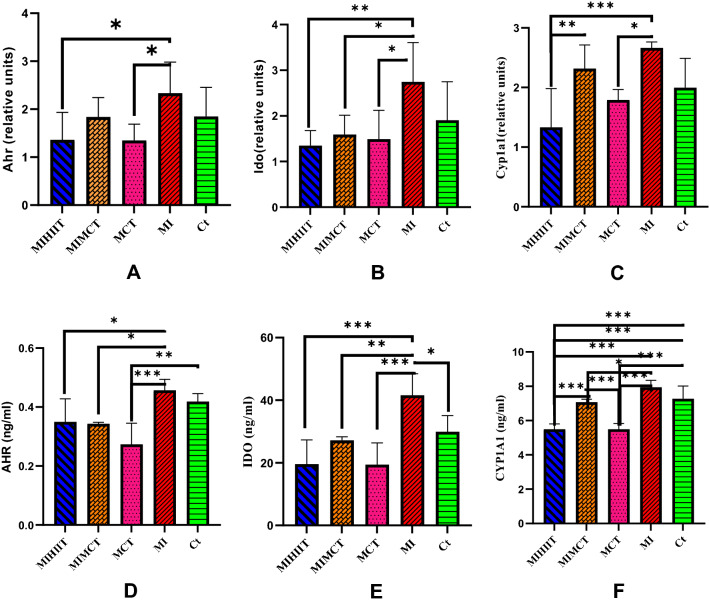
*Ahr* (**A**), *Ido1* (**B**), and *Cyp1a1* (**C**) gene expression, and AHR (**D**), IDO1 (**E**), and CYP1A1 (**F**) protein levels in the heart tissue of rats. Gene expressions and protein levels were increased in the MI condition and training showed decreasing effects in them. Abbreviations: *Ct* control group, *MI* the group with occlusion of the left coronary artery, *MICT* the group treated with moderate intensity of continuous training, *MIMCT* rats with left coronary artery occlusion treated with moderate intensity of continuous training, *MIHIIT* rats with left coronary artery occlusion treated with high-intensity interval training.

The results in Fig. 2, indicated that the MI increased the mRNA levels of *Ahr*, *Ido1*, and *Cyp1a1* compared to the control group, but this increase was not statistically significant. Also, the changes in proteins level were not significant except for IDO1, and only levels of this protein significantly (P < 0.027) increased in the MI group in comparison to Ct rats (Fig. 2F). Both training protocols significantly reduced the levels of AHR, IDO, and CYP1A1 proteins in MIHIIT and MIMCT groups, compared with the MI group (P < 0.001), in which HIIT training had a greater effect than MICT. In healthy male rats, only protein expression of AHR significantly (P < 0.05) decreased in the MICT group compared to the control group (Fig. 2D). Both HIIT and MICT training protocols significantly reduced the *Cyp1a1* and *Ido1* expressions and CYP1A1 and IDO1 proteins levels in heart tissue, and a greater effect of HIIT was observed (Fig. 2B,C,E,F).

### Effects of interventional factors on variables

Box’s M-test showed the observed covariance matrices of the dependent variables are equal across groups (P = 0.064). The significant effect of the group on the latent variables (AHR, CYP1A1, KYN, IDO1, and MDA simultaneously) was observed (Wilks’ λ = 0.027, Partial Eta Square = 0.60, P < 0.001). Considering the significant effect on the latent variable, individual univariate analysis was done and showed that there is a significant effect of group on variables as AHR (F_5,42_ = 10.32, P < 0.001), CYP1A1 (F_5,42_ = 37.71, P < 0.001), KYN (F_5,42_ = 10.36, P < 0.001), IDO1 (F_5,42_ = 13.52, P < 0.001) and MDA (F_5,42_ = 12.70, P < 0.001).

According to Table 3A, based on the **r** coefficient there was a highly positive significant correlation between the studied factors. Also, in the bivariate analysis, there was a moderate positive correlation between factors (based on the **r**_**p**_ coefficient), however, it was significant just for some of them (Table 3A). The results of the Sidak post hoc test and pairwise comparison of groups are presented in Figs. 1 and 2.

Table 3B presented the parameter estimates of variables that the control group was considered as a reference and other groups were compared to it. This test showed that MI resulted in the elevation of all variables and HIIT and MICT significantly reduced these factors levels and only in the CYP1A1 variable, MICT couldn’t exert a significant effect. It seems that between the two types of exercise, HIIT had better performance (Table 3B).

## Discussion

The evidence has confirmed that exercise training can improve cardiac function in heart diseases such as MI and improve the quality of life in patients. Although for the early exercise rehabilitation following MI, resistance training and MICT have been proposed as beneficial choices^18^ supervised HIIT was reported to result in greater improvements in MI patients^16^. A randomized controlled study found that aerobic interval training increases peak oxygen uptake more than usual exercise training in myocardial infarction patients^19^. It has been shown that aerobic exercise with moderate intensity could improve physical capacity and other cardiovascular variables^26^. However, HIIT has shown a relatively low rate of major adverse cardiovascular events for patients with coronary artery disease or heart failure when applied within cardiac rehabilitation settings^17^. Exercise intensity is an important factor for reversing left ventricular remodeling and improving aerobic capacity, endothelial function, and quality of life in patients with postinfarction heart failure^27^.

The results of the present study showed that both training protocols significantly reduced the level of KYN in the heart tissue of rats with MI, however, the effect of HIIT was greater than MICT (Fig. 1A). KYN is a new and valuable biomarker of chronic heart failure, with the ability to predict mortality and reflect exercise capacity^28^. Impairment of heart rhythm and observations of myocardial cell failure induced by KYN have been reported earlier and elevated plasma levels of KP metabolites and their ratios are associated with increased mortality, independent of coronary artery disease, in patients with heart failure^10,29^. Considering that KYN metabolites may increase inflammation, oxidative stress, and apoptosis of smooth muscle cells and endothelial cells it has been suggested that alterations of tryptophan metabolism might have an impact on the bioenergetic activities of heart mitochondria and might be involved in the development of clinical symptoms such as cardiomyopathy^8,30,31^. KYN generation through IDO is markedly induced after MI and genetic deletion or pharmacological inhibition of IDO limits cardiac injury and cardiac dysfunction after MI^9^, and here we showed that exercise reduces KYN levels in heart tissue significantly.

Exercise-induced KP modulates energy homeostasis and may contribute to the prevention and treatment of chronic diseases. Physical exercise has been shown to impact the KP in response to both acute and chronic exercise training stimuli^12^. Physical exercise can modulate KP metabolism in skeletal muscle and thus change the concentrations of select compounds in peripheral tissues and the central nervous system^14^. The effects of KP metabolites on skeletal muscle, adipose tissue, the immune system, and the brain suggest that some of these compounds could qualify as exercise-induced myokines^14^. Endurance exercise training can change KP metabolites by changing the levels of KP enzymes in skeletal muscle. This leads to a metabolite pattern that favors energy expenditure and an anti-inflammatory immune cell profile and reduces toxic metabolites^32^.

MI significantly increases MDA levels in heart tissue and here we showed that MICT and HIIT significantly reduced the levels (Fig. 1B). MDA is a known oxidative stress marker for coronary artery disease severity and plaque sensitivity^33^ and its reduction is considered to be an indicator of the healing of myocardial ischemia injuries^34^.

Also, the AHR pathway has been reported to exert cardioprotective effects against cardiotoxicity and produce heart-specific transcriptional responses^35,36^. KYN induces cardiomyocyte apoptosis through reactive oxygen species production in an AHR–dependent mechanism^9^. The expression level of circulating AHR may affect the susceptibility and progression of coronary arterial disease^37^ and it participates in myocardial ischemia–reperfusion injury by regulating mitochondrial apoptosis^38^. The results of the present study showed that OLAD resulted in an increase of *Ahr* gene expression in the heart tissue of rats with MI and MICT decreased it but none of these changes were statistically significant. However, at the protein level, both training protocols significantly reduced the level of AHR in the heart tissue of rats with OLAD. Additionally, even in healthy controls, MICT reduced the level of AHR protein in cardiac tissue. In general, both training protocols were able to reduce the AHR protein level in heart tissue. Previous studies have reported that in the myocardial ischemia model with OLAD, AHR is abundantly expressed in necrotic myocardium and it is shown that acute myocardial ischemia can activate AHR and induce inflammation^39^. Also, some animal studies reported a decrease in AHR after improvement of cardiac condition^40^. It seems that some AHR ligands, such as BaiCalin1, could reduce myocardial necrosis and inflammation by inhibiting the cardiac expression of AHR^38,41^. The heart and its vasculature system express all AHR-regulated genes and cardiac AHR-regulated CYPs are involved in the pathogenesis of cardiovascular diseases^42^. Increased gene expression of *Cyp1a1* with MI was not statistically significant. However, in comparison to MICT HIIT reduces *Cyp1a1* expression significantly. Regarding protein levels, both exercise protocols significantly reduce CYP1A1, although the effect of HIIT was greater (Fig. 2C,F). Parameter Estimation in Table 3 shows that among protein expressions HIIT had the greatest effect and only in CYP1A1, MICT training was most effective. It has been clearly shown that cardiac AHR-regulated CYPs are involved in the pathogenesis of cardiovascular diseases^42^.

In the present study, MI increased the level of *Ido1* gene expression but it was not statistically significant. Both types of HIIT and MICT significantly reduced the *Ido1* gene expression in heart tissue, however, HIIT had a greater effect and the same changes were observed for IDO1 protein levels (Fig. 2B and E). IDO1 and the IDO1-associated pathway constitute critical mediating agents associated with immunoinflammatory responses such as atherosclerosis in the heart tissue^43^. IDO1 promotes cardiomyocyte hypertrophy partially via PI3K-AKT-mTOR-S6K1 signaling^44^ and its suppression could potentially reduce the inflammatory response in cardiomyocyte injury^45^. IDO1 was inversely associated with ischemic heart disease with a directionally consistent estimate for stroke and might be a potential therapeutic target for this disease^11^. Based on the results in Table 3A, the protein expression of the studied variables is significantly correlated with each other. Also, it is shown that these variables are significantly related and affected by each other. A decrease in AHR led to a decrease in IDO1 and IDO1 had a significant relationship with KYN and CYP1A1 (Table 3A).

It is reported previously that HIIT was more effective than moderate-intensity training for improving oxygen pulse (O_2_P) slope in coronary heart disease patients, while ventilation and carbon dioxide production (VE/VCO_2_) slope and oxygen uptake efficiency slope were similarly improved by aerobic training regimens versus controls^46^. A systematic review and meta-analysis reported that HIIT is superior to MICT in improving cardiorespiratory fitness in participants of cardiac rehabilitation^47^. Our results are somehow consistent with the finding of these studies, however, there were some limitations. Measuring the slopes related to oxygen physiology, analyzing the activity of the enzymes involved in the oxidative/antioxidative system in heart tissue, assessing other components of the *Ido1*-Kyn-*Ahr* axis and immunohistochemical studies could add highly valuable data to future studies on this topic.

In conclusion, based on this study, it is possible to conclude that myocardial infarction alters the *Ido1*-Kyn-*Ahr* axis in heart tissue cells and imbalances it, as well as triggering oxidative stress. Both high-intensity interval training and moderate-intensity continuous training were effective at reducing the levels of the axis components and HIIT had a more significant effect. The intensity of exercise appears to be a prominent factor in ameliorating this molecular axis in cells of the infarcted heart.

## Data Availability

The datasets used and/or analysed during the current study available from the corresponding author on reasonable request.

## Abbreviations

AhR: Aryl hydrocarbon receptor
Ct: Control group
HIIT: High-intensity interval training
IDO1: Indoleamine 2,3-dioxygenase 1
KYN: Kynurenine
KP: KYN pathway
LAD: Left anterior descending
MDA: Malondialdehyde
MICT: Moderate-intensity continuous training
MANOVA: Multivariate analysis of variance
MI: Myocardial infarction
OLAD: Occluded LAD
MIHIIT: Rats with OLAD and treated with HIIT
MIMCT: Rats with OLAD and treated with MICT
